# The mapping of cancer incidence and mortality trends in the UK from 1980–2013 reveals a potential for overdiagnosis

**DOI:** 10.1038/s41598-018-32844-x

**Published:** 2018-10-02

**Authors:** Jason L. Oke, Jack W. O’Sullivan, Rafael Perera, Brian D. Nicholson

**Affiliations:** University of Oxford, Nuffield Department of Primary Care Health Sciences, Oxford, OX2 6GG United Kingdom

## Abstract

The incidence of cancer in the United Kingdom has increased significantly over the last four decades. The aim of this study was to examine trends in UK cancer incidence and mortality by cancer site and assess the potential for overdiagnosis. Using Cancer Research UK incidence and mortality data for the period (1971–2014) we estimated percentage change in incidence and mortality rates and the incidence-mortality ratio (IMR) for cancers in which incidence had increased >50%. Incidence and mortality trend plots were used to assess the potential for overdiagnosis. Incidence rates increased from 67% (uterine) to 375% (melanoma). Change in mortality rates ranged from −69% (cervical) to +239% (liver). The greatest divergences occurred in uterine (IMR = 132), prostate (IMR = 9.6), oral (IMR = 9.8) and thyroid cancer (IMR = 5.3). Only in liver cancer did mortality track incidence (IMR = 1.1). For four cancer sites; uterine, prostate, oral and thyroid, incidence and mortality trends are suggestive of overdiagnosis. Trends in melanoma and kidney cancer suggest potential overdiagnosis and an underlying increase in true risk, whereas for cervical and breast cancer, trends may also reflect improvements in treatments or earlier diagnosis. A more detailed analysis is required to fully understand these patterns.

## Introduction

In the UK, it is estimated that one in two people will develop cancer during their lifetime, a figure that at the turn of the century was one in three^1^. Large increases in the incidence have been seen in many but not all cancer types whilst overall cancer mortality has decreased^2^. There are a number of possible explanations for the continued increases. Undoubtedly, the UK’s ageing population^3^ is a contributing factor as cancer is predominately a disease of old age. Increased exposure to risk factors for cancer such as smoking^4^, alcohol^5^, UV exposure^6^, HPV infection^7^, and chronic diseases such as obesity^8^ and Type 2 diabetes^9^ are potential drivers of incidence. Yet neither the aging population nor increases in exposure to risk can explain all of the significant changes in cancer incidence. A recent study suggested that only a third of all cancers are attributed to modifiable risk factors^10^ and even after accounting for the confounding effect of age through standardisation^11^ incidence has increased dramatically.

**Figure 1:**
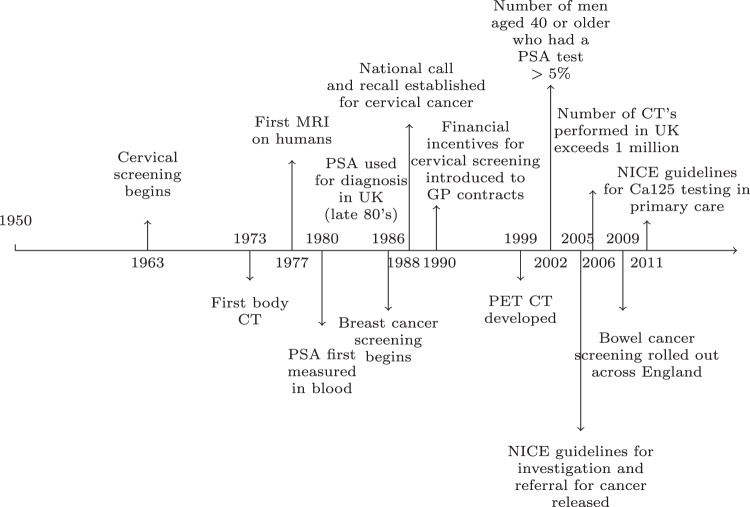
Timeline showing important “milestones” in cancer diagnostic practice in the UK.

An alternative explanation is that increased incidence is due to the change in diagnostic practice. The increase in incidence has coincided with introduction of population wide cancer screening programmes, increased use of diagnostic tests for case-finding, widening disease definitions^12^ and widespread use of advanced imaging technology^13^ (Fig. 1). These initiatives are intended to diagnose cancer early, increasing the opportunity for curative management and in turn improving prognosis and preventing death from cancer. However, there is also a growing body of evidence suggesting that these early detection initiatives tend to uncover cancers that were previously missed or ignored. These pseudo-cancers are not false positives^14^ as they meet the definitions of cancer but they are cancers that regress without treatment, are indolent and never produce symptoms, or grow so slowly that the patient dies of another cause. For cancers of the thyroid, prostate and breast, autopsy studies have shown that the reservoir of pseudo-cancers is substantial^15^.

Randomised control trials of screening for prostate, breast, and lung cancer have repeatedly demonstrated that these cancers can be found and manifest as an excess of cancers in screened arms of trials, that persist even with extensive follow-up^16–18^. As it is not possible to know which cancers these are, patients may be subjected to unnecessary and potentially harmful treatments, whilst finite health resources are wasted.

**Figure 2:**
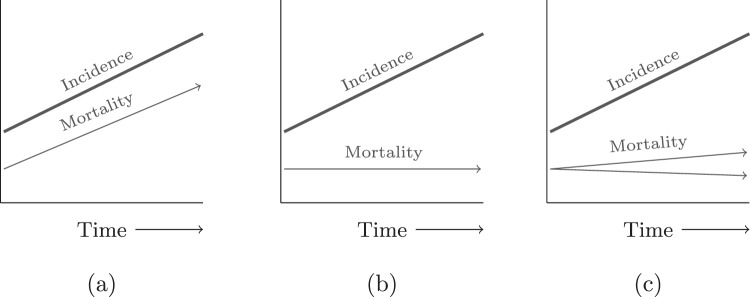
Trends in incidence and mortality for (**a**) a true increase in disease and (**b**) a disease being overdiagnosed and (**c**) overdiagnosis predominates but changes in underlying disease risk.

Welch and Black^19^ suggested that the presence of overdiagnosis at a population level can be seen in the way incidence and mortality changes over time. They argued that if there had been true increase in life threatening and clinically important cancer, mortality trends would follow increases in incidence over time. Similarly, they argued that if rapidly rising rates of diagnoses coincided with a stable mortality trends then overdiagnosis was highly likely. Recent analysis of thyroid cancer has suggested a third way with two underlying processes in which the dominant one is overdiagnosis and the other being a small but real increase in incidence^20^. Therefore, we consider three distinct patterns of mortality trends in the presence of rising incidence (see Fig. 2). Whilst overdiagnosis has been described most frequently in populations with organised cancer screening, evidence is also accumulating for drivers of overdiagnosis outside of screening and outside of cancer: widening disease definitions, recommendations to investigate for cancer at lower risk thresholds, and the incidental detection of abnormalities in patients being scanned for other reasons^21^.

There has been no report describing trends in incidence and mortality rates with respect to overdiagnosis using UK-wide cancer data. With this in mind, the aim of this study was to examine trends in UK cancer incidence trends in relation to mortality trends over the last four decades.

## Methods

We obtained European age-standardised cancer-specific incidence data from Cancer Research UK for England, Wales and Scotland (GB) for the period 1979–2013, incidence data for the UK (GB + Northern Ireland) for the period 1993–2014 and UK mortality data for the period 1971–2014^22^. Incidence and mortality figures were standardised to the 2013 version of the European Standard Population (ESP). The standardisation across time removes the effect of shifting age demographics over time.

A comparator population is required to detect overdiagnosis in populations with a stable or even declining incidence rate. In the absence of comparator data we focussed on the cancer sites with increase in incidence in excess of 50% to establish if overdiagnosis could be present.

We calculated the percentage change in incidence and mortality between the baseline (1980) and study end period (2013) by subtracting the 3-year average of years 2012–14 from the average for 1979–81 and dividing by the 1979–81 average. We summarised the difference in the change in incidence and mortality by calculating the absolute percentage change in incidence divided by the absolute change in mortality between the study end and the baseline period for each cancer site, the incidence-mortality ratio (IMR). Confidence intervals for the IMR were calculated using a bootstrap resampling method.

We plotted the total incidence and mortality trends over time, separating invasive cancer incidence from total cancer incidence (*in-situ* + invasive) and invasive for cervical and breast cancer. Figures were plotted on the log scale so that proportionality could be assessed, i.e. if incidence and mortality increased at similar rates then we would expect to see parallel lines when these are plotted on a log-scale.

We compared trends in cancer incidence and mortality rates to assess for patterns corresponding to either: (A) increased incidence and mortality in the same spectrum of disease, (B) increased incidence and stable mortality driven by the detection of previously undetected indolent disease (C) increased incidence and decreased mortality driven by the detection of indolent disease and the effective treatment of disease detected early that would otherwise have caused death. In the absence of any validated method to objectively categorise incidence and mortality trends we have used qualitative judgment in order classify individual cancer sites into A, B or C types.

### Transparency declaration

The lead author (JO) affirms that the manuscript is an honest, accurate, and transparent account of the study being reported; that no important aspects of the study have been omitted; and that any discrepancies from the study as planned (and, if relevant, registered) have been explained.

## Results

### Incidence and mortality trends

The incidence of 10 of the 20 most common cancers in the UK has increased by more than 50% in both sexes since the 1980’s. These are cancers of the breast, cervix, kidney, liver, melanoma, non-Hodgkin lymphoma (NHL), oral, prostate, thyroid, and uterine cancers. The largest relative change in incidence is observed for melanoma, increasing by 375% (Table 1). The incidence of cervical, kidney, liver, NHL, prostate and thyroid cancer have all increased by more than 150% since 1980. The lowest relative change was in uterine cancer which has increased 67% over the same period (Table 1). For breast, cervical, thyroid and uterine cancer, mortality was lower at the end of the study period compared to the start. The largest relative reduction in cancer specific mortality was in cervical cancer, which has reduced by 69% since 1980. The only other cancer to see significant reductions in cancer mortality was breast (35% reduction in mortality). In all but liver cancer, the change in incidence has far exceeded the change in mortality.

**Table 1:** European age-standardised GB incidence and UK mortality rates (per 100,000) for ten cancers for 1980 to 2013 in men and women, for women only* and men only**. Figures for baseline (1980) and follow-up (2013) based on three-year averages for that period (1979–1981 and 2012–2014). *** 2013 estimate of incidence based on three-year average from 2011–13. IMR - incidence to mortality ratio. Significance tests for a change in deaths since baseline are all *p* < 0.001 except ^†^*p* = 0.012 and ^††^ = 0.76.

Cancer site		New cases (per 100,000)			Deaths (per 100,000)			IMR (95% CI)
		1980	2013	% change	1980	2013	% change	
Breast*	*In-situ* (D05)	3	23	+580	55	36	−35	2.4 (2.1 to 2.6)
	Invasive (C50)	101	169	+67				
	*In-situ* + Invasive	105	192	+84				
Cervical*	*In-situ* (D06)	20	92	+356	9	3	−69	2.5 (1.8 to 3.7)
	Invasive (C53)	18	10	−44				
	*In-situ* + Invasive	38	102	+169				
Kidney and other urinary tract (C64–66,C68)		7	20	+180	5	7	+60	3.0 (2.6 to 3.3)
Liver (C22)		3	9	+257	2	8	+239	1.1 (0.9 to 1.2)
Melanoma (C43)		5	25	+375	2	4	+93	4.0 (3.3 to 4.7)
Non-Hodgkin Lymphoma (C82–85)		9	23	+162	5	8	+51	3.2 (2.8 to 3.8)
Oral (C00-06, C09-C10,C12–14)***		7	13	+92	4	4	+9^†^	9.8 (6.6 to 19.2)
Prostate (C61)**		69	179	+157	42	48	+16	9.6 (6.4 to 11.7)
Thyroid (C73)		2	5	+165	1	0.7	−31	5.3 (4.1 to 6.2)
Corpus uteri and uterus NOS (C54-C55)*		18	29	+67	7	7	−1^††^	132 (4.1 to 226.0)

### Invasive vs *in-situ* cancer

**Figure 3:**
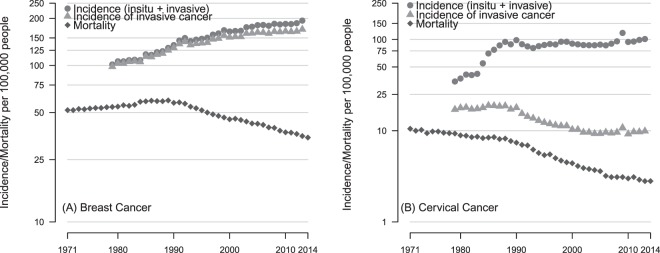
European age-standardised incidence (GB) and cancer-specific mortality (UK) per 100,000 women for (**A**) breast cancer (ICD 10 codes C50 and D05) and (**B**) cervical cancer (C53 and D06).

Increases in invasive breast cancer account for the majority of breast cancers but the incidence of *in-situ* forms has increased by over 500% from 3 per 100,000 in 1980 to 23 per 100,000 in 2013 (Fig. 3). Breast cancer incidence rose quickly between 1980 and 1999 at 2.6% per year then slowed to 1%. The decline in breast cancer mortality followed three years after the introduction of the breast screening programme in 1986 (See Fig. 1).

*In-situ* forms of cervical cancer (cervical intra-epithelial neoplasia (CIN III), adenocarcinoma *in-situ*, intraepithelial glandular neoplasia and severe dysplasia of cervix uteri) dominate the incidence of cervical cancer and account for all of the increase as the rates of invasive cancer have almost halved since 1980. Cervical cancer incidence, driven entirely by *in-situ* forms of the disease rose dramatically after 1983 at >22% per year to but has since slowed to 1.9% per year. The sharp peak in *in-situ* and invasive cancer diagnoses around 2009 is likely to be a result of the well-publicised diagnosis of a British reality TV star Jade Goody^23^. Mortality from cervical cancer declined rapidly from the beginning of the study period and continues to decline at a rate of 1.7% per year. Breast and cervical with their increasing incidence but declining mortality resemble type “C” patterns.

### Potential for overdiagnosis?

**Figure 4:**
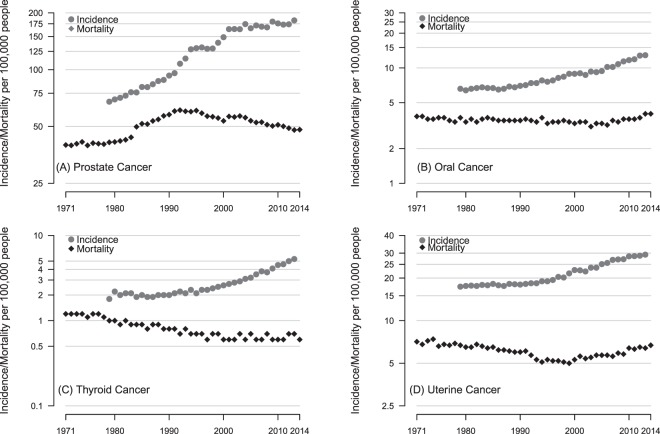
European age-standardised Incidence and cancer-specific mortality (per 100,000) for (**A**) prostate (males only), (**B**) oral (males and females), (**C**) thyroid (males and females) and (**D**) uterine cancer (females only).

**Figure 5:**
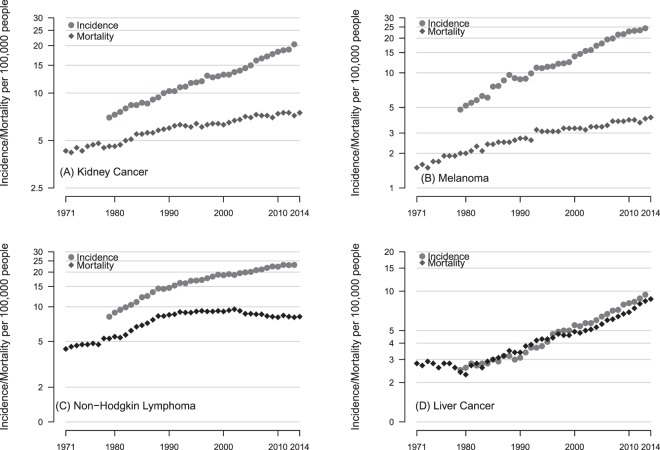
European age-standardised Incidence and cancer-specific mortality (per 100,000) for (**A**) kidney, (**B**) melanoma, (**C**) NHL and (**D**) liver cancer. Figures are for men and women combined.

For four cancer sites; prostate, thyroid, oral and uterine, the incidence rate increased more than 5 times faster than the mortality rate. In thyroid, oral and uterine cancers the mortality has not changed significantly in over three decades, for prostate cancer mortality increased then declined but is yet to return to where the level seen in the early seventies. These four cancers bear the closest resemblance to the classic type “B” pattern of increasing incidence and stable mortality (Fig. 4). The trends in liver cancer closely resemble the type “A” pattern but kidney cancer, melanoma and NHL are “C” type with mortality increasing but not nearly to the extent of the increases in incidence (see Fig. 5).

## Discussion

Our analysis highlights that the incidence of ten of the most common cancers in the UK has increased significantly over the last four decades at a higher rate than cancer specific mortality by several orders of magnitude. Only liver cancer mortality increased in line with incidence. For the remaining cancer sites a complex relationship can be seen between incidence and mortality. Four main mechanisms could explain increased cancer incidence without a corresponding increase in cancer specific mortality, which are discussed hereafter in relation to the UK’s health system.

Indolent disease is more likely to be detected in asymptomatic populations if a widely available means of detection and a large disease reservoir is present. National breast, colorectal and cervical cancer screening programmes operate in the UK and whilst the UK does not have a screening programme for prostate cancer, PSA testing can be requested in primary care. PSA testing became widely available in the UK in the late 1980’s (see Fig. 1) and may explain much of the rise in new diagnoses in this period. At the end of the nineties, there is a plateauing (of incidence) and this may have been in response to two systematic reviews which clearly stated that there was insufficient evidence to recommend mass-screening for prostate cancer as a public health policy^24^.

Stage migration or diagnostic drift is evidenced by disproportionate increases in *in-situ* or early-stage cancers and has been observed regionally in the UK^25^, the Netherlands^26^, and the US^27^ for melanoma. Awareness of skin cancer is high in the UK. The public are encouraged to check their moles through public awareness campaigns such as Be Clear on Cancer and avoid waiting to see a doctor by having them checked by private high-street clinics. The scale and impact of private screening, high-street clinics (individual health assessments), work schemes, and charity initiatives on stage migration and diagnostic drift is largely unknown. However, it has been shown that early borderline lesions may be categorised as malignant to avoid the consequences of the misdiagnosis of more aggressive cancers^28^.

Early diagnosis initiatives may advance the diagnosis of progressive cancers to such an extent that the person dies from other causes first (competing risks). Cancers with long pre-clinical phases diagnosed late in life are particularly prone to this form of overdiagnosis. For example, average lead-times for PSA detected prostate cancers are in the region of 4.5 to 12 years with median of 7 years in the UK. Even if one assumed that all prostate cancers detected by PSA were progressive, 20% of men will die of another cause if diagnosed aged 70–74 rising to 50% in men aged 85 to 89^29^.

The number of NHS CT scans rose from less than 2 million in 1995 to in excess of 5 million in 2013 and a 9% increase is currently projected per year^30^. Kidney cancer is particularly prone to incidental detection as advanced imaging modalities are able to detect very small abnormalities unlikely to be related to the symptoms that warranted the test^31^. The contribution of incidental findings to the total number of kidney cancer diagnoses in the UK is unknown but they could make up a significant proportion as the most common form renal cell carcinoma is commonly diagnosed as an incidental finding^32^.

### Comparison with existing literature

To our knowledge, this is the first study to analyse cancer incidence and mortality trends using data from the UK and examine trends across a range of cancer sites. Global trends in breast cancer incidence and mortality have varied widely^33^. Our study has found patterns similar to those described in the U.S. for melanoma, prostate, breast, and kidney cancer^19^. Patterns of increasing incidence and stable or increasing mortality have also been observed for prostate cancer across Europe. We report smaller increases in thyroid cancer detection than those seen in South Korea following the introduction of ultrasound screening^34^ and those recently reported from the US^20^ In these data, oral and uterine cancer exhibit pattern of incidence and mortality that could resemble overdiagnosis, but very little has been described about this in the literature. Oral cancer is commonly “screened” for by dentists in the UK but evidence is limited for its effectiveness. A recent United States Preventative Service Task Force (USPSTF) evidence review found one RCT of oral cancer screening; the Trivandrum Oral Cancer Screening Study. Screening every three years for a maximum of three rounds led to an extra 47 cancers in the screening arm of the trial (rate ratio = 1.16, 95% C.I. 0.7 to 1.92)^35^. Patterns of uterine or *endometrial* cancer detection could have arisen through changes in diagnostic practices, in particular in which atypical endometrial hyperplasia is now labelled as endometrial cancer^36^.

Could the observed increases in cancer be simply due to cancers shifting from being labeled as “cancer of unknown primary” or CUP to specific cancer sites? Since 1993, there has been a significant drop (50%) in the incidence of CUP from 32 per 100,000 in 1993 to 15 per 100,000 in 2015 for all persons. The reduction in the incidence of CUP has been attributed to improvements in data collection and diagnostic capabilities which mean fewer cases registered as CUP’s and more being identified by primary site^37^. This would result in an increase in more new cases. However, as mortality from CUP has decreased by 45% over the same time period, any increase in incidence would be matched by an concomitant increase in mortality rates. Moreover, the scale of the reduction in CUP is much smaller than the increases seen for many of the cancers we have examined in this study.

### Limitations

Our analysis has several limitations. Firstly, we did not have access to incidence by stage or histological sub-type for all cancers. Historically, coverage of this level of data in the UK has been incomplete and has not been integrated into national statistics databases. Disproportionate increases in the early stages of cancer are suggestive of overdiagnosis in the two cancers for which we have data (cervical and breast cancer) we observe a substantial increase in the early stage forms. Secondly, we used incidence statistics for both Great Britain and the UK, but mortality rates from just the UK. This is because although UK mortality data is available from 1971, UK incidence statistics only go back to 1993. We also present data for oral cancer incidence only up to 2013 as latest figures have expanded the definition whereas mortality data is consistent with previous definitions. Sensitivity analyses show how closely matched UK and GB estimates of incidence are (see appendix 1). Some may question our choice of baseline period: we took the earliest possible years for incidence (1979) and mortality (1971). We could have chosen a more recent baseline period but this would have diminished the scale of change and coincided with introduction of diagnostic practice that we suspect has driven the increases (Fig. 1). The classification system we have used extends the idea first proposed by Welch and Black^19^ from two to three categories. Even with three patterns, this still represents a simplification of what are complex processes that have the potential to evolve over time. An example of this is evident for NHL which in the first half of the study resembles a type A and the second half looks like a type B pattern (see Fig. 5).

### Implications

In the UK, the last four decades have seen marked increases in cancer incidence which is rarely matched by similar scale increases in cancer related mortality. We suggest overdiagnosis could be a significant contributor to these trends. Whilst the risks of harm may be small in absolute terms they may quickly erode the benefit of early detection: there are many more people at risk of overdiagnosis than people with aggressive cancer. For some cancers, these risks are being acknowledged, for example in prostate cancer there has been a shift towards watchful waiting in low-risk localised cancers^38^, and trials of surveillance first strategies are ongoing to avoid aggressive management in ductal carcinoma *in-situ*(DCIS)^39^. Results of these trials may offer strategies to limit the risks of overdiagnosis together with high-level calls to reclassify low-grade carcinomas as atypical or indolent lesions.

## Conclusions

We have identified cancers for which the potential for overdiagnosis has not been previously described in the UK. As early diagnosis initiatives receive increasing support, investment must be directed towards improving our understanding of which cancers need to be diagnosed and which of these need to be treated.

## Electronic supplementary material

Supplementary information

## Data Availability

The datasets generated during and/or analysed during the current study are available from the corresponding author on reasonable request.

## References

[1] Quinn, M., Babb, P., Brock, A. & Kirb, L., J. Cancer trends in England and Wales 1950–199. Tech. Rep., Office for National Statistics (2000).

[2] Oke J L, Nicholson B D, Shinkins B (2015). Comment on: ‘Trends in the lifetime risk of developing cancer in great Britain: comparison of risk for those born from 1930 to 1960’—cancer predictions need more context. British Journal of Cancer.

[3] Ahmad A S, Ormiston-Smith N, Sasieni P D (2015). Trends in the lifetime risk of developing cancer in Great Britain: comparison of risk for those born from 1930 to 1960. British Journal of Cancer.

[4] Vineis P (2004). Tobacco and Cancer: Recent Epidemiological Evidence. Journal of the National Cancer Institute.

[5] Allen NE (2009). Moderate alcohol intake and cancer incidence in women. Journal of the National Cancer Institute.

[6] Parkin DM, Boyd L, Walker LC (2011). 16. The fraction of cancer attributable to lifestyle and environmental factors in the UK in 2010. British journal of cancer.

[7] zur Hausen H (2009). Papillomaviruses in the causation of human cancers— a brief historical account. Virology.

[8] Renehan AG, Tyson M, Egger M, Heller RF, Zwahlen M (2008). Body-mass index and incidence of cancer: a systematic review and meta-analysis of prospective observational studies. The Lancet.

[9] Yang W-S (2016). Type 2 diabetes and the risk of non-Hodgkin’s lymphoma. European Journal of Cancer Prevention.

[10] Tomasetti C, Vogelstein B (2015). Variation in cancer risk among tissues can be explained by the number of stem cell divisions. Science.

[11] Kirkwood, B. & Sterne, J. *Essential Medical Statistics, eTextbook BT - 123Library*, 2 edn (Wiley-Blackwell, 2010).

[12] Moynihan R, Doust J, Henry D (2012). Preventing overdiagnosis: how to stop harming the healthy. BMJ.

[13] Black WC, Welch HG (1993). Advances in diagnostic imaging and overestimations of disease prevalence and the benefits of therapy. The New England journal of medicine.

[14] Brodersen J (2018). Overdiagnosis: what it is and what it isn’t. BMJ Evidence-Based Medicine.

[15] Furuya-Kanamori L, Bell KJL, Clark J, Glasziou P, Doi SAR (2016). Prevalence of differentiated thyroid cancer in autopsy studies over six decades: A meta-analysis. Journal of Clinical Oncology.

[16] Marcus PM (2006). Extended Lung Cancer Incidence Follow-up in the Mayo Lung Project and Overdiagnosis. JNCI Journal of the National Cancer Institute.

[17] Miller AB (2014). Twenty five year follow-up for breast cancer incidence and mortality of the Canadian National Breast Screening Study: randomised screening trial. BMJ.

[18] Schröder FH (2009). Screening and prostate-cancer mortality in a randomized European study. The New England Journal of Medicine.

[19] Welch HG, Black WC (2010). Overdiagnosis in cancer. Journal of the National Cancer Institute.

[20] Lim H (2017). Trends in Thyroid Cancer Incidence and Mortality in the United States, 1974–2013. JAMA.

[21] Nicholson B.D. (2017). Detecting cancer in primary care: Where does early diagnosis stop and overdiagnosis begin?. European Journal of Cancer Care.

[22] Cancer Research UK Cancer statistics by type, http://www.cancerresearchuk.org/health-professional/cancer-statistics/statistics-by-cancer-type (2016).

[23] Lancucki L, Sasieni P, Patnick J, Day TJ, Vessey MP (2012). The impact of Jade Goody’s diagnosis and death on the NHS Cervical Screening Programme. Journal of Medical Screening.

[24] Bryant, R. & Hamdy, F. Trends in Prostate Cancer Screening: Overview of the UK. In D. P. Ankerst *et al*. (ed.) *Prostate cancer screening*, 15–23 (Humana Press, 2009).

[25] Shuster S (2009). Malignant melanoma: how error amplification by screening creates spurious disease. The British journal of dermatology.

[26] van der Leest RJT (2015). Increasing time trends of thin melanomas in The Netherlands: What are the explanations of recent accelerations?. European journal of cancer (Oxford, England: 1990).

[27] Welch HG, Woloshin S, Schwartz LM (2005). Skin biopsy rates and incidence of melanoma: population based ecological study. BMJ (Clinical research ed.).

[28] Levell NJ, Beattie CC, Shuster S, Greenberg DC (2009). Melanoma epidemic: a midsummer night’s dream?. The British journal of dermatology.

[29] Pashayan N, Powles J, Brown C, Duffy SW (2006). Excess cases of prostate cancer and estimated overdiagnosis associated with PSA testing in East Anglia. British journal of cancer.

[30] Cancer Research UK & 2020 Delivery HORIZON SCANNING. An evaluation of imaging capacity across the NHS in England, https://www.cancerresearchuk.org/sites/default/files/horizon_scanning_-_final.pdf (2015).

[31] Kihira T, Shiraishi T, Yatani R, Roa I, Liu PI (1991). Pathological features of renal cell carcinoma incidentally discovered at autopsy. Acta pathologica japonica.

[32] Siegel CR (2015). Incompletely characterized incidental renal masses: Emerging data support conservative management. Journal of Urology.

[33] Héry C, Ferlay J, Boniol M, Autier P (2008). Quantification of changes in breast cancer incidence and mortality since 1990 in 35 countries with Caucasian-majority populations. Annals of oncology: official journal of the European Society for Medical Oncology/ESMO.

[34] Ahn HS, Kim HJ, Welch HG (2014). Korea’s Thyroid-Cancer “Epidemic”— Screening and Overdiagnosis. New England Journal of Medicine.

[35] Sankaranarayanan R (2005). Effect of screening on oral cancer mortality in Kerala, India: a cluster-randomised controlled trial. The Lancet.

[36] Oda K (2016). Risk of endometrial cancer in patients with a preoperative diagnosis of atypical endometrial hyperplasia treated with total laparoscopic hysterectomy. Gynecology and Minimally Invasive Therapy.

[37] Cancer Research UK Cancer of unknown primary incidence statistics, http://www.cancerresearchuk.org/health-professional/cancer-statistics/statistics-by-cancer-type/cancer-of-unknown-primary/incidence#heading-Two (2016).

[38] Loeb S (2017). Active Surveillance Versus Watchful Waiting for Localized Prostate Cancer: A Model to Inform Decisions. European Urology.

[39] Francis A (2015). Addressing overtreatment of screen detected DCIS; the LORIS trial. European journal of cancer (Oxford, England: 1990).

