# Supplementary material for: The mapping of cancer incidence and mortality trends in the UK from 1980–2013 reveals a potential for overdiagnosis
Source: Sci Rep. 2018 Oct 2;8:14663. doi: 10.1038/s41598-018-32844-x (PMC6168593; doi:10.1038/s41598-018-32844-x)
Supplement: Supplementary file 1 — Supplementary information [file 41598_2018_32844_MOESM1_ESM.pdf]

The mapping of cancer incidence and mortality trends in the UK from 1980-2013 reveals a potential for overdiagnosis.

Jason L. Oke, Jack W. O'Sullivan, Rafael Perera and Brian D. Nicholson

University of Oxford, Nuffield Department of Primary Care Health Sciences, Oxford, OX2 6GG, United Kingdom

Supplementary files

Appendix 1: Sensitivity analysis comparing UK and GB incidence data

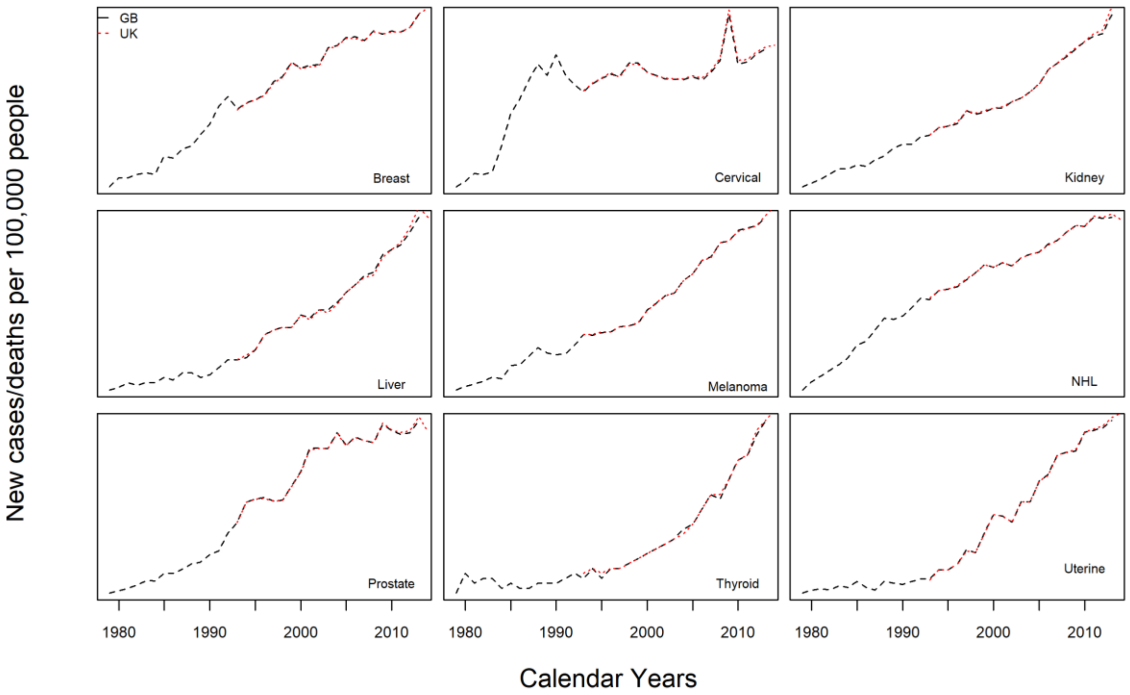

Appendix 2: Strobe statement

STROBE Statement—checklist of items that should be included in reports of observational studies

	Item No	Recommendation
**Title and abstract**	1	(*a*) Indicate the study's design with a commonly used term in the title or the abstract<br><br>*The title makes it clear that this is an analysis of trends and the abstract clearly states that this done using UK incidence and mortality data*
		(*b*) Provide in the abstract an informative and balanced summary of what was done and what was found<br><br>*See Abstract*
**Introduction**		
Background/rationale	2	Explain the scientific background and rationale for the investigation being reported<br><br>*From the section "what this paper adds"*<br><br>*There has been a significant increase in the risk of developing cancer in the UK and across the world. At the same time, the total number of deaths from cancer has declined. When the number of new cases increase sharply whilst cancer mortality remains stable over the same time period, overdiagnosis is suspected. Cancer overdiagnosis is an unintended consequence of cancer screening, advanced imaging, and other early detection methods.. Until now, no such study has used UK data to examine the potential for overdiagnosis across cancer types with and without organised screening.*<br><br>*What this study adds.*<br><br>*Over recent decades the UK has seen trends in incidence and mortality rates that resemble but do not strictly cohere with the patterns associated*

		with overdiagnosis. As screening programmes expand and early diagnosis initiatives develop, investment is urgently required to understand how to mitigate to harms of overdiagnosis whilst improving cancer outcomes further.
Objectives	3	State specific objectives, including any prespecified hypotheses<br><br>*In abstract:*<br><br>*To examine trends in cancer incidence and mortality in the UK between 1980 and 2013 and to assess whether overdiagnosis may explain current trends.*
**Methods**		
Study design	4	Present key elements of study design early in the paper<br><br>*Key elements of design are in first two paragraphs of methods section*
Setting	5	Describe the setting, locations, and relevant dates, including periods of recruitment, exposure, follow-up, and data collection<br><br>*First two paragraphs of methods section*<br><br>*Setting : UK and Great Britain (England, Scotland and Wales).*<br><br>*Relevant dates: 1979 to 2013.*<br><br>*Exposures: NA*<br><br>*Follow-up: NA*<br><br>*Data collection: We used cancer-specific mortality and incidence data from the Cancer Research UK website*
Participants	6	Give the eligibility criteria, and the sources and methods of selection of participants. Describe methods of follow-up<br><br>*"People diagnosed with and dying from cancer in Great Britain."*
Variables	7	Clearly define all outcomes, exposures, predictors, potential confounders, and effect modifiers. Give diagnostic criteria, if applicable

		Outcome measures are listed in abstract and methods section paragraph subtitled Stage 1 and Stage 2<br><br>"we estimated percentage change in incidence and mortality rates for the most common cancer sites in which incidence had increased > 50%. Plots showing incidence and mortality trends as well as the absolute ratio of change in incidence to mortality (IMR) was used to assess divergence between incidence and mortality trends".
Data sources/ measurement	8*	For each variable of interest, give sources of data and details of methods of assessment (measurement). Describe comparability of assessment methods if there is more than one group<br><br>*Incidence and mortality statistics were taken from Cancer research UK website*
Bias	9	Describe any efforts to address potential sources of bias<br><br>*As this is analysis is descriptive rather than hypothesis driven we feel the risk of bias is minimal here. It is possible that we could be over-fitting segments to incidence and mortality trends. In order to minimise this we have always fitted sought models with the least number of breakpoints.*
Study size	10	Explain how the study size was arrived at<br><br>*N/A - data is from national statistics.*
Quantitative variables	11	Explain how quantitative variables were handled in the analyses. If applicable, describe which groupings were chosen and why:<br><br>*Primary quantitative variable, incidence and mortality were natural log-transformed when used in regression models.*
Statistical methods	12	(*a*) Describe all statistical methods, including those used to control for confounding<br><br>*See methods section:*

(*b*) Describe any methods used to examine subgroups and interactions

In a previous version of this analysis we analysed trendsin men and women separately.

(*c*) Explain how missing data were addressed

This is described in the methods section.

"We obtained age-standardised cancer-specific incidence data from Cancer Research UK for England, Wales and Scotland (GB) for the period 1979 -2013, incidence data for the UK (GB + Northern Ireland) for the period 1993-2014 (25) and UK mortality data for the period 1971-2014. Incidence and mortality figures were standardised to the 2013 version of the European Standard Population (ESP). The standardisation across time removes the effect of shifting age demographics over time. Weights for the age-standardisation method are given in appendix 1."

(*d*) *Cohort study*—If applicable, explain how loss to follow-up was addressed

NA

(*e*) Describe any sensitivity analyses

Previous analyses used simple linear regression in place of segmented regression. We have not included this data in the paper but the results did not affect our conclusion.

Continued on next page

Results

Participants	13*	(a) Report numbers of individuals at each stage of study—eg numbers potentially eligible, examined for eligibility, confirmed eligible, included in the study, completing follow-up, and analysed<br><br>*N/A - numbers we used were rates*
		(b) Give reasons for non-participation at each stage: *N/A*
		(c) Consider use of a flow diagram: N/A
Descriptive data	14*	(a) Give characteristics of study participants (eg demographic, clinical, social) and information on exposures and potential confounders<br><br>*N/A Population statistics so data not available at individual level*
		(b) Indicate number of participants with missing data for each variable of interest<br><br>*N/A*
		(c) *Cohort study*—Summarise follow-up time (eg, average and total amount)<br><br>*N/A*
Outcome data	15*	*Cohort study*—Report numbers of outcome events or summary measures over time<br><br>*N/A*
		Case-control study—Report numbers in each exposure category, or summary measures of exposure<br><br>*N/A*
		Cross-sectional study—Report numbers of outcome events or summary measures<br><br>*N/A*
Main results	16	(*a*) Give unadjusted estimates and, if applicable, confounder-adjusted estimates and their precision (eg, 95% confidence interval). Make clear which confounders were adjusted for and why they were included<br><br>No confounders or explanatory variables were used in this study.<br><br>.
		(*b*) Report category boundaries when continuous variables were categorized:<br><br>*N/A*

		(*c*) If relevant, consider translating estimates of relative risk into absolute risk for a meaningful time period<br><br>*N/A*
Other analyses	17	Report other analyses done—eg analyses of subgroups and interactions, and sensitivity analyses<br><br>*N/A*
**Discussion**		
Key results	18	Summarise key results with reference to study objectives<br><br>*Paragraph 1 of discussion section.*
Limitations	19	Discuss limitations of the study, taking into account sources of potential bias or imprecision. Discuss both direction and magnitude of any potential bias<br><br>*See sub-section titled Limitations*
Interpretation	20	Give a cautious overall interpretation of results considering objectives, limitations, multiplicity of analyses, results from similar studies, and other relevant evidence<br><br>*This has been done see Discussion section especially limitations and comparison to the literature sections*
Generalisability	21	Discuss the generalisability (external validity) of the study results<br><br>*See discussion*
**Other information**		
Funding	22	Give the source of funding and the role of the funders for the present study and, if applicable, for the original study on which the present article is based

This study was not funded directly but JLO, RP, BN and JOS all receive funding from the National Institute for Health Research (NIHR).

*Give information separately for cases and controls in case-control studies and, if applicable, for exposed and unexposed groups in cohort and cross-sectional studies.

Note: An Explanation and Elaboration article discusses each checklist item and gives methodological background and published examples of transparent reporting. The STROBE checklist is best used in conjunction with this article (freely available on the Web sites of PLoS Medicine at http://www.plosmedicine.org/, Annals of Internal Medicine at http://www.annals.org/, and Epidemiology at http://www.epidem.com/). Information on the STROBE Initiative is available at www.strobe-statement.org.

Appendix 3: Table shows the 2013 ESP standard ages and weights.

Table 1: European standard population ages and weights

Age Group (years)	Standard Population
0,0	1,000
1-4	4,000
5-9	5,500
10-14	5,500
15-19	5,500
20-24	6,000
25-29	6,000
30-34	6,500
35-39	7,000
40-44	7,000
45-49	7,000
50-54	7,000
55-59	6,500
60-64	6,000
65-69	5,500
70-74	5,000
75-79	4,000
80-84	2,500
85-89	1,500
90-94	800
95+	200
**Total**	**100,000**

Source: Eurostat
